# Is Optometry the Practice of Medicine?

**Published:** 1913-01

**Authors:** F. Park Lewis


### Is Optometry the Practice of Medicine?

By F. PARK LEWIS, M.D.

DOES the adaptation of glasses for the relief of symptoms constitute the practice of medicine, or does it not? If it does, it is the work of the physician. If it does not it is the work of the mechanician.

The question involved is not one having to do with the technical difficulties of glass fitting, because adequate training in optics and practical work in refraction will make a capable man an adept without any knowledge whatever of the anatomy of the human eye or of the intricasies of medicine. On the other hand no matter how complete may be an education in medicine it will not make a skilled refractionist until he shall have been trained in the physics of light in its effects upon the human eye.

It is necessary only to understand how profound is this influence to realize that this is not only not work for a mere mechanician, but it often demands for its successful application the widest knowledge, the broadest medical training, and the most acute and logical reasoning on the part of the trained physician. We are only now beginning to understand that *light* is one of the essentials of *life,* and that through the eyes by means of a most complex series of adjustments it produces actual changes in the cortex of the brain which make us conscious of the outside world. Let us disarrange these sensitive adjustments, as we may at any time do experimentally by placing prisms, or spherical lenses of unequal strength, or wrongly adjusted cylinders before the eyes, and we can produce symptoms of illness as immediate and as profound as those which would result from a dose of a toxic drug. It is now known that the changes of color in the chameleon, affecting as they do the entire body, are produced through the sympathetic nervous system by way of the eyes, and when the eyes of the animal are destroyed, no such alterations of color are produced. It is within the experience of every careful ophthalomolgist that the endeavor to blend the images when the eyes are organically unlike will often result in nausea and vertigo, while a cold sweat and a profound exhaustion, are the indications of a real sickness which the confusion of sight produces. There is no careful internist who will be satisfied that his treatment is complete, especially in a neurotic patient, until he is assured that the eyes are working in harmony and without strain. He knows how multiform are the reflexes carried by the long bundle of nerve fibres extending from the nucleus of the 3rd nerve, under the fourth ventricle, until they are lost in the medulla. He has seen chorea relieved by the cure of hyperphoria and has witnessed the disappearance of psychosis coincidently with the relief of a muscle strain. It is no longer a subject of doubt but a matter of daily observation that the eyes through the brain are the most prolific source of general disturbances. With normal vision the eye ground may indicate serious constitutional disease. The condition of the retina may give the first evidence of lesions in the kidney, in which the promptitude of recognition may mean the saving of life. Rapid increase of presbyopia is an early symptom of glaucoma, and the early diagnosis of a glioma in the eyes of a child (so like a cataract to the untrained) will save him from the horrors of a cancer involving his entire head. To recognize these the refractionist must be a physician.

On the other hand the doctor who undertakes to prescribe for eyes must be adequately trained, not only in the physics of light, but in the exceedingly difficult work of refraction. This cannot be learned in a few weeks nor can it be academically acquired by a course in a correspondence school. It must be patiently studied in the clinic under the eye of a good teacher. It is by no means a mere mechanical matter. After having determined the refractive value of each eye, both in the long and short range of vision, having ascertained the muscle balance, having eliminated the latent conditions which are frequently found in the most difficult cases, having determined whether the marginal lid irritation present may not be due to some nasal obstruction or some bodily habit, he arrives at the most difficult and most important point, to which all of this leads, the logical treatment which must follow. Shall it be the local application of some medicinal substance to the lids?—or the internal administration of a drug? or the use by hypodermatic injection of one of the sera which will raise the opsonic index ? or the relief from ciliary strain by correcting refractive errors, or by the use of a prism? or should the use of the eyes temporarily cease, and the eyes be given rest? The right prescription of glasses requires fully as discriminating judgment as does a determination of the static refraction, and the experienced ophthalomologist will never follow a rule of thumb. His success in the most difficult cases will depend on the skill with which he departs from established usage. To be able to do this satisfactorily requires a most careful and extended training in ophthalmology as well as in medicine. It is not to be wondered at, therefore, that men who have been graduated in medicine in a course in which ophthamology has been merely touched upon, and who yet have the temerity to undertake to practice a specialty which has grown into a distinctive branch of the profession, make refractive blunders which "though they make the unskilled laugh cannot but make the judicious grieve."

In an analysis of the situation there are two objects to be considered—the best interests of the patient which are necessarily first, and for whose benefit both optician and ophthalomologist exist, and just and fair treatment of both the latter. To attain the former there should be an official assurance of competency on the part of those who treat the eye whether with glasses, drugs, or what not. This could be determined by the issuance of a degree in ophthalmology given only after demonstrated proficiency. A little learning is not only dangerous but misleading. The refractionist must first be a doctor. He need not be an expert in abdominal surgery nor in orthopaedy, but he should learn the interrelation of the part with the whole, as of the whole to the part. Per contra, is it too much to exact that the optometrist shall be a mechanic as well as a physicist? In their struggle to be quasi doctors very few of the opticians have taken the trouble to acquire any degree of proficiency in the work which primarily is expected of them, i. e., the making of glasses so that they shall be properly ground and correctly set, and the adjustment of frames so that they shall easily, firmly and comfortably maintain their position on the face. The skilled making of lenses is a profession which has before now attracted the attention of men of the best ability. It demands for its successful prosecution a knowledge of optics and of mathematics. In a complicated prescription it is of first importance that the refractive indices of the lenses combined shall be known in order that the effect which they shall produce, may be calculated in advance. In the decentering of a lens a prism element is introduced the effect of which must be estimated. The work of the optician is therefore wholly different from that of the optometrist.

Can the latter without a knowledge of brain physiology measure the visual field and draw logical deductions from his findings? Can he as the most expert and well trained ophthalmologist can not, always determine the true refraction without the use of a cycloplegic? Can he, without the use of a mydriatic discover the beginnings of cataract in the lens margin? Can he, without some pathological experience be assured that the redness of the lids is due to eye strain and not to the infectious and dreaded trachoma? If he cannot, must not his examinatiion at best, be a partial and misleading one giving an unwarranted sense of security to the credulous patient who is quite unable to measure the medical limitations of one who choses to call himself a "Doctor of Optometry?" Is it too much to hope that all of those who profess to treat the eyes whether doctors of medicine or optometrists, shall be required by the state to give evidence of a practical and theoretical knowledge of the subject in all of its branches so that at least the minimum requirement of "reasonable knowledge and skill" shall be met?

It would seem that an *entente* could be effected by which not a *little* medicine but a *real* medical training shall be the basis of the education of the refractionist and that the maker of lenses shall be at least as well equipped for this work as is the dispenser of drugs for his.

